# A chimeric poxvirus with *J2R* (thymidine kinase) deletion shows safety and anti-tumor activity in lung cancer models

**DOI:** 10.1038/s41417-019-0114-x

**Published:** 2019-06-17

**Authors:** Shyambabu Chaurasiya, Nanhai G. Chen, Jianming Lu, Nikolas Martin, Yinan Shen, Sang-In Kim, Susanne G. Warner, Yanghee Woo, Yuman Fong

**Affiliations:** 1 Department of Surgery, City of Hope National Medical Center, Duarte, CA USA; 2 Ottawa Hospital Research Institute, Ottawa, Canada; 3 Department of Hepatobiliary and Pancreatic Surgery, The Second Affiliated Hospital, Zhejiang University School of Medicine, Hangzhou, China

**Keywords:** Non-small-cell lung cancer, Gene therapy

## Abstract

Oncolytic viruses have shown excellent safety profiles in preclinical and clinical studies; however, in most cases therapeutic benefits have been modest. We have previously reported the generation of a chimeric poxvirus (CF33), with significantly improved oncolytic characteristics, through chimerization among different poxviruses. Here we report the sequence analysis of CF33 and oncolytic potential of a GFP-encoding CF33 virus (CF33-GFP) with a *J2R* deletion in lung cancer models. Replication of CF33-GFP and the resulting cytotoxicity were higher in cancer cell lines compared to a normal cell line, in vitro. After infection with virus, cancer cells expressed markers for immunogenic cell death in vitro. Furthermore, CF33-GFP was safe and exerted potent anti-tumor effects at a dose as low as 1000 plaque forming units in both virus-injected and un-injected distant tumors in A549 tumor xenograft model in mice. Likewise, in a syngeneic model of lung cancer in mice, the virus showed significant anti-tumor effect and was found to increase tumor infiltration by CD8+ T cells. Collectively, these data warrant further investigation of this novel chimeric poxvirus for its potential use as a cancer bio-therapeutic.

## Introduction

Lung cancer is the second most common cancer type in both men and women [1]. Despite advancements in diagnosis and treatment lung cancer remains the leading cause of cancer mortality worldwide [2]. For early stage disease, surgery is the first line therapy whereas for advanced, unresectable disease a combination of chemo- and radiation is commonly used [3–5]. Rate of recurrence after initial remission is high for lung cancer and 5-year survival is dismal at 18% [1]. Hence, alternative therapeutics need to be developed for better treatment of lung cancer.

Oncolytic viruses (OVs) are replication-competent viruses that can exert anti-tumor effects directly *via* lysis of cancer cells, and indirectly *via* activation of immune system [6]. A wide range of viruses, either in their natural forms or engineered, have been studied for their oncolytic properties and several of them have entered clinical trials [7]. Recently, the first OV Talimogene laherparepvec (T-VEC), a herpes simplex virus encoding GM-CSF was approved by the FDA for treatment of melanoma [8]. Another OV in advanced clinical trials is Pexa-Vec, a vaccinia virus (VACV) encoding GM-CSF [9]. While most of the OVs including T-VEC and Pexa-Vec have demonstrated excellent safety profile in clinical settings, their anti-tumor efficacy has been modest so far [10–13]. Hence, there is a need for developing new OVs with enhanced efficacy without compromising the safety.

Recent studies have shown that death of cancer cells may be immunogenic or tolerogenic [14]. Immunogenicity of a dying cell depends mainly on three damage-associated molecular patterns: surface expression of calreticulin, release of adenosine triphosphate (ATP), and release of high mobility group box 1 (HMGB1) [15, 16]. Interestingly, several studies suggest that overall efficacy of cancer therapeutics depends on their ability to induce an anti-tumor immune response [17, 18]. Hence, immunogenic death of cancer cells, which would elicit anti-tumor immunity, is preferred over non-immunogenic cell death. Many types of OVs have been reported to induce the markers of immunogenic cell death (ICD) in cancer cells [19].

We previously reported the construction of a chimeric poxvirus (CF33), with enhanced anti-tumor activity, through chimerization among 9 species of orthopoxvirus including multiple strains of VACV [20, 21]. Here, for the first time we report the genetic sequence analysis of the CF33 virus. Furthermore, we report the oncolytic properties of a CF33 derivative that has the *J2R* (thymidine kinase) gene replaced with a GFP expression cassette (CF33-GFP) in lung cancer. We tested cancer specificity of CF33-GFP in vitro using cancer and normal cell lines. Next, we investigated whether the virus could induce markers for ICD in lung cancer cells. Finally, we examined safety and oncolytic efficacy of the virus in xenograft and syngeneic mouse models of lung cancer.

## Materials and methods

### Cell lines

African green monkey kidney cells CV-1, human lung cancer cells A549, H1299, H1650 and mouse lung cancer cells LLC1 as well as human dermal fibroblast HDFa were purchased from ATCC (Manassas, USA). A549, H1299, and H1650 were cultured in RPMI-1640. CV-1, LLC1, and HDFa were grown in DMEM. All media were supplemented with 10% fetal bovine serum (FBS), 2 mM L-glutamine and 100 U/ml penicillin-streptomycin. Media and supplements were purchased from Corning (Corning, NY, USA). Cells were maintained in a humidified incubator at 37 °C and 5% CO_2_.

### Virus genome analysis

Genome of CF33 was sequenced using next generation sequencing. The genomic sequence of CF-33 was aligned with the sequences of VACV strains Ankara (AS, accession number: U94848), Western Reserve (WR, NC_006998), IHD-W clone IHDW1 (KJ125439), Lister (KX061501), cowpox strain Brighton Red (AF482758), rabbitpox strain Utrecht (AY484669), and raccoonpox strain Herman (NC_027213) using Kalign multiple sequence alignment [22]. Based on the obtained alignment, genomic regions without gaps in the alignment and with high identity to any parental strain were identified. Identity (99.8% or higher) to the parental genomes was confirmed using Basic Local Alignment Search Tool (BLAST®). The map of the genome was created using SnapGene® Viewer.

### Construction of CF33-GFP

CF33 was generated through chimerization among multiple species/strains of poxviruses as described previously [20]. To construct CF33-GFP, a cDNA encoding Emerald (GFP) under the H5 early/late promoter was inserted into CF33 at the *J2R* locus. Briefly, GFP expression cassette with the VACV H5 promoter was PCR-amplified from the plasmid Emerald-pBAD (Addgene, Cambridge, MA). The PCR fragment was digested with *Sac*I and *Bam*HI and cloned into plasmid p33NC-TK to yield p33NCTK-GFP. CV-1 cells were infected with CF33 at a multiplicity of infection (MOI) of 0.1 for 1 h and then transfected with p33NCTK-GFP using jetPRIME transfection reagent (Polyplus-transfection Inc.). Two days post-infection, cells were harvested and recombinant viruses were selected and plaque purified as described previously [23].

### Virus proliferation and cytotoxicity assays

The ability of viruses to replicate in cultured cells was determined by plaque assays. Cells were infected at an MOI of 0.03 and virus titers in the lysates were determined by standard plaque assay as described previously [21]. CellTiter 96® AQ_ueous_ colorimetric Assay (Promega) was used for measuring cell survival following virus infection. Briefly, cells were infected in 96-well plates at indicated MOIs. CellTiter 96® AQ_ueous_ was added 72 h post-infection and absorbance was measured at 490 nm using a plate reader (Tecan Spark). Survival was calculated relative to mock-infected wells.

### Flow cytometry

Cells were mock-infected or infected with *TK*-deleted CF33 virus at MOI 5 in a 6-well plate. The relatively high MOI (MOI 5) was used to ensure that all cells get infected by the virus. Eighteen hours post-infection, cells were detached using 10 mM EDTA and stained with AlexaFluor488-conjugated anti-calreticulin antibody (ab196158; Abcam) or isotype antibody (ab199091; Abcam). Cells were fixed with 4% paraformaldehyde and analyzed on a flow cytometer (Accuri C6; BD).

### Western blot analysis

Cells were infected with CF33-GFP virus at MOI 5 in a 6-well plate. Medium from wells were collected at 24, 48, and 72 h and concentrated 10 folds using centrifugal filters (UFC801024; Amicon). Next, 25 μL of each sample was separated on a Criterion Precast polyacrylamide gel (3450028; BIO-RAD) then transferred to a PVDF membrane. The membrane was stained with anti-HMGB1 antibody (ab18256; Abcam) and donkey anti-rabbit antibody (926–32213; Li-COR) in iBind Flex FD solutions (1930520; Invitrogen) using iBind Flex Western Device (ThermoFisher) for 3 h. The membrane was then scanned using Azure C600 scanner (Azure Biosystems).

### Determination of secreted ATP

Cells were infected with CF33-GFP at MOI 5 in a 6-well plate for 1 h. After 1 h, inoculum was aspirated and 1 mL of medium containing 0.5 % FBS was added per well. Medium from each well was collected 18 h post-infection and levels of ATP in the medium were determined using luminescence-based ATP determination kit (A22066; Molecular Probes) according to the manufacturer’s protocol.

### Animal studies

For xenograft model, 4–6 weeks old athymic female mice *(*Envigo, USA) were acclimatized for 1 week and injected with 5 × 10^6^ A549 cells (mixed 1:1 in matrigel) sub-cutaneously on both sides of the flank. For syngeneic model, 4–6 weeks old C57BL/6 mice (Charles River, USA) were acclimatized and injected with 5 × 10^4^ LLC1 cells (mixed 1:1 in matrigel) sub-cutaneously into lower flank, one tumor/mouse.

Weight and tumor volumes were measured twice weekly. When tumors reached an approximate volume of 150 mm^3^ (A549) or 50 mm^3^ (LLC1), mice were sorted into treatment groups. Next, mice were injected with PBS or CF33-GFP. For A549 tumors, 1000 plaque-forming units (PFUs) of virus was injected only once in the right-side tumor. For LLC1 model, tumors were injected on day 1, 3, and 5 (10^7^ PFUs/injection). Mice were euthanized when volume of a tumor exceeded 2500 mm^3^.

To assess bio-distribution of viruses, mice treated as above were euthanized on day 7 and 56 after treatment for A549 model and on day 7 for LLC1 model. Tumors and organs were harvested, homogenized and virus titers were determined by standard plaque assay.

All animal studies were conducted under a City of Hope Institutional Animal Care and Use Committee approved protocol in compliance with National Institute of Health’s guideline for care and use of laboratory animals.

### In vivo imaging

Mice were imaged for GFP using small animal imaging equipment (LagoX imaging system) and images were processed using the AMIview image processing software. Mean fluorescence intensity (MFI) of GFP signal was calculated for each tumor at different time points using the AMIview image processing software.

### Immunohistochemical analysis

Tumors were harvested, formalin fixed, paraffin-embedded and sectioned. Sections were de-paraffinized followed by heat-mediated antigen-retrieval. Sections were blocked for 20 min with TNB Blocking buffer (PerkinElmer), and then incubated with rabbit anti-CD8alpha (ab209775; Abcam) in TNB blocking buffer overnight. Next day, sections were washed and incubated with HRP-conjugated goat anti-rabbit (ab6721; Abcam) for 1 h. Brown color was developed using DAB (cat#ab64238; Abcam), and the sections were counterstained with hematoxylin (MHS16; Sigma-Aldrich). Images were taken with Zeiss Z1 Axiobserver microscope (Carl Zeiss Microscopy). CD8+ T cells in tumor sections were counted using image Pro software (Media Cybernetics).

### Statistical analysis

Statistical analyses comparing two groups were performed using Student’s *t*-test. For comparisons of more than two groups, one-way ANOVA was used. *P*-values ≤ 0.05 were deemed significant. Survival studies were analyzed for statistical significance using the log-rank Mantel-Cox test. GraphPad Prism 5 (GraphPad Software, La Jolla, CA) was used to calculate statistical values.

## Results

### Genomic analysis of CF33

The genomic sequence of CF33 was aligned with the sequences of VACV strains Ankara (177,923 bp), Western Reserve (194,711 bp), IHD-W clone IHDW1 (195,821 bp), Lister (187,893 bp), cowpox strain Brighton Red (224,499 bp), rabbitpox strain Utrecht (197,731 bp) and raccoonpox strain Herman (214,699 bp) using Kalign multiple sequence alignment [22]. Out of the nine parental viruses, sequence alignment was performed only with seven viruses whose sequences were publicly available. VACV CL and VACV Lederle-Chorioallantoic (LC) were not used for alignment, as their sequences were not available. Based on the obtained alignment, genomic regions without gaps in the alignment and with high identity (99.8% or higher) to any parental strain were identified.

**Fig. 1:**
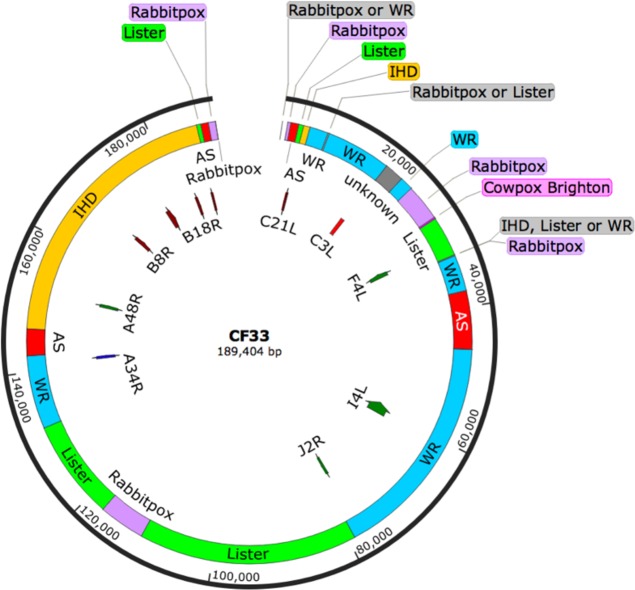
Genomic analysis of CF33. Map of virus genome showing components of parental viruses. Origin of genes involved in host immune-modulation (C21L, C3L, B8R, and B18R) and nucleotide metabolism (F4L, I4L, J2R, and A48R) are also shown

Sequence analysis of CF33 revealed that the virus genome is derived mostly from three strains of VACV: IHD, WR and Lister (Fig. 1). About 3 kb of CF33 genome (16,244–19,120 bp; labeled ‘unknown’ in Fig. 1) did not match with any of the seven parental viruses, these sequences may have come from VACV CL and/or VACV LC. Interestingly, no sequence from Raccoonpox virus was detected in CF33 genome.

We determined the origin of some genes that play crucial role in modulating host immunity or are important for the replication of the virus in non-dividing cells. The products of *C21L* and *C3L* genes are complement-binding proteins that inhibit antibody-dependent complement-enhanced neutralization of virus [24, 25]. In CF33 virus, *C21L* and *C3L* are derived from Lister and WR strains of VACV, respectively. The products of *B18R* and *B8R* are soluble proteins that act as decoy receptors for type I and type II interferons, respectively [26, 27]. Both *B8R* and *B18R* in CF33 have their origin in IHD strain of VACV. Likewise, in CF33 virus the gene *A34R*, encoding a glycoprotein required for the infectivity of extracellular-enveloped virus [28], originates from WR strain of VACV. Products of the genes *J2R, F4L, I4L*, and *A48R* are all involved in the synthesis of nucleotides and these genes are important for virus replication in non-dividing cells with low levels of nucleotides [29]. In CF33, *J2R*, and *I4L* have their origin in WR strain of VACV whereas *F4L* and *A48R* originate from Lister and IHD strains, respectively. In summary, in CF33 virus most of the genes crucial for evading host cell’s immunity or generating nucleotides for viral DNA synthesis are derived from one of the 3 VACV strains: IHD, WR or Lister.

### CF33-GFP shows cancer-specific growth and cytotoxicity in vitro

**Fig. 2:**
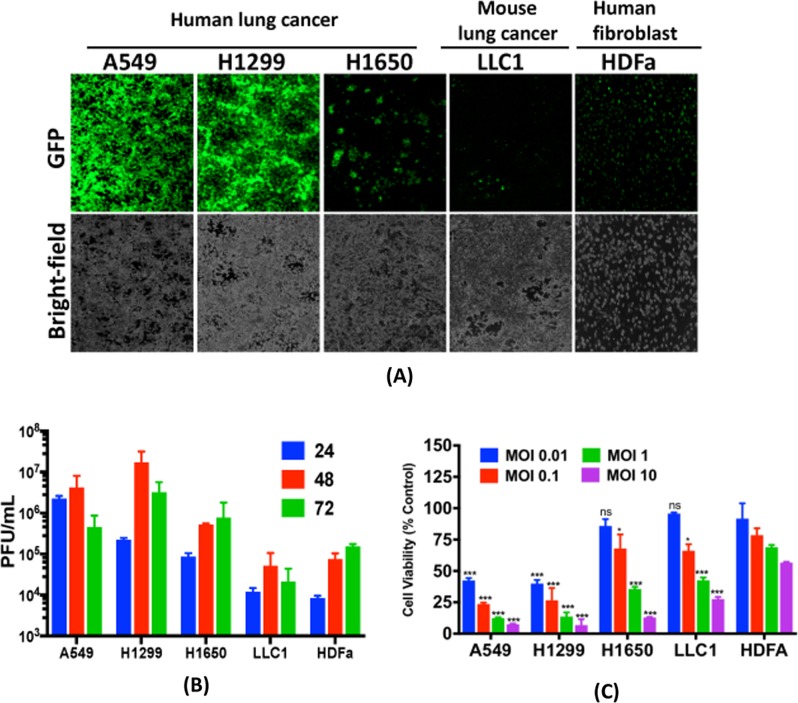
CF33-GFP preferentially replicates in and kills cancer cells. **a** Human lung cancer cells (A549, H1299 and H1650), mouse lung cancer cells (LLC1) and human fibroblast cells (HDFa) were infected with CF33-GFP at MOI of 0.03. Forty-eight hours post-infection, images were taken using a fluorescent microscope. **b** Cells infected as in (**a**) were harvested at indicated time points and virus titers in the harvested cell lysates were determined using standard plaque assay. **c** Cells were infected with the virus at indicated MOIs and cell survival relative to mock-infected cells was calculated 72 h post-infection. Data shown as mean ± SD. All experiments were repeated at least twice. Stats: two-way ANOVA comparing cell viabilities at different MOIs in HDFa cell line with those in other cell lines. *P < 0.05, **P < 0.01, ***P < 0.005

CF33-GFP was used to infect human lung cancer cells (A549, H1299 and H1650), mouse lung cancer cells (LLC1) and human fibroblast (HDFa). Cells were imaged for virus-encoded GFP using fluorescent microscope. Intensity of GFP in human lung cancer cells was much higher compared to that in the fibroblast, HDFa (Fig. 2a). Furthermore, growth of the virus and resulting cytotoxicity were higher in human lung cancer cells compared to those in HDFa (Fig. 2b, c). However, in mouse cancer cells LLC1, the virus was found to be less effective, which is not surprising as several studies in past have shown that mouse cancer cells are less responsive to VACV [30, 31] (Fig. 2b, c).

### CF33-GFP infection induces markers of immunogenic death in cancer cells

**Fig. 3:**
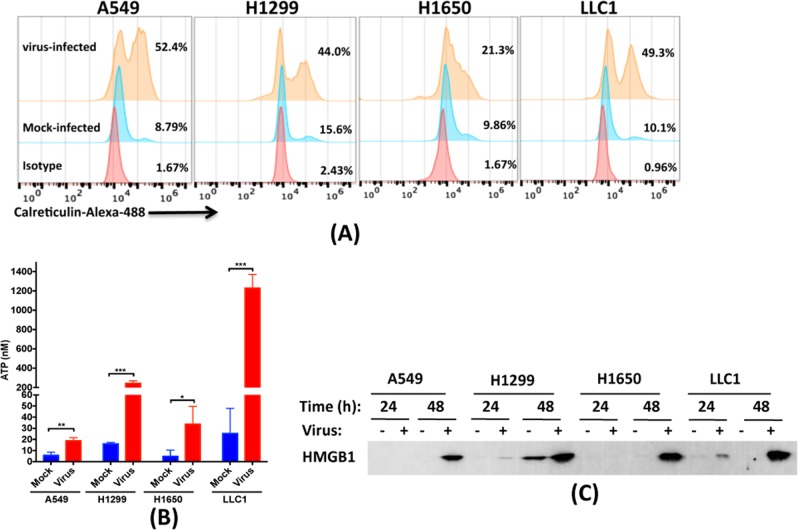
CF33 induces markers of immunogenic cell death. **a** Cells were infected with *TK*-deleted CF33 virus. Eighteen hours post-infection, cells were detached and stained for calreticulin and analyzed by flow cytometry. For this particular experiment we used CF33 that is deleted of TK but not encoding GFP, since the antibody we used was labelled with Alexa488. **b** Cells were infected with the CF33-GFP or mock-infected, and ATP released in the medium was quantified 18 h post-infection. Each bar represents mean ± SD (*n* = 3). Stats: student’s *t*-test; **P < 0.01, ***P < 0.005. **c** Cells were infected at MOI 5 and medium from virus or mock-infected cells were collected at indicated time points. HMGB1 was detected in the collected medium using Western blot analysis

To determine if CF33-GFP induces ICD in lung cancer cells, we infected cells with virus and tested for aforementioned markers of ICD. Compared to the mock infection, all the cancer cell lines were found to increase surface expression of calreticulin and release ATP after virus infection (Fig. 3a, b). Likewise, high level of HMGB1 was released in the medium, especially at later time point (48 h), after infection of cells with the virus (Fig. 3c). These results are consistent with our previously published study where we used colorectal cancer model [21].

### CF33-GFP efficiently spreads to distant tumors in a mouse model of human lung cancer

**Fig. 4:**
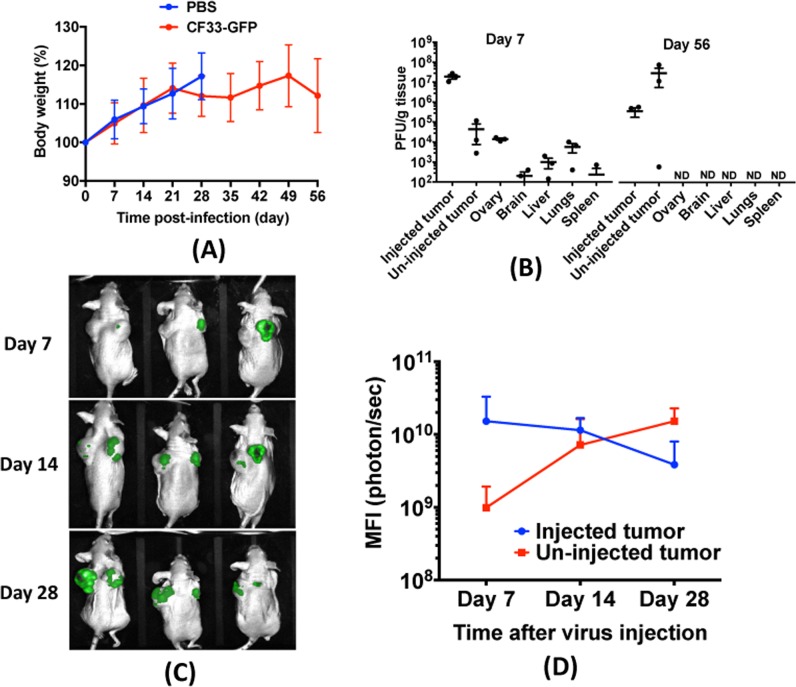
CF33-GFP is safe in mice. Nude mice bearing bilateral sub-cutaneous A549 xenografts were injected intra-tumorally with 10^3^ PFUs of CF33-GFP or PBS only in the right-sided tumors. **a** Mice were weighed weekly and percent body weight was calculated. Data pooled from two independent experiments (*n* = 5 + 5 per group) and presented as mean ± SD. **b** In a parallel experiment, virus-injected mice (*n* = 3) were euthanized on day 7 and 56 after treatment. Virus titer in tumors and normal organs were determined by standard plaque assay. ND: not detected; Limit of detection: 100 pfu/g. **c** Mice were imaged for green fluorescence weekly and images were processed using the AMIview image processing software. **d** Mean fluorescence intensity (MFI) for virus-injected and un-injected tumors were calculated and compared. Data shown as mean ± SD

Immune-compromised mice bearing bilateral A549 xenografts in their flanks were injected intra-tumorally with 10^3^ PFUs of CF33-GFP or PBS in only one of the two tumors. Mice were monitored for sign of toxicity and were weighed twice weekly. No overt sign of toxicity was observed, and all virus-treated mice continued gaining weight similar to PBS-treated mice (Fig. 4a). In a parallel experiment,three mice were euthanized on day 7 and 56 after virus injection, and virus titers in organs and tumors were determined. While high titers of the virus were detected in tumors at both time points, in normal organs virus was detected only at day 7, albeit at least 3-log lower than in tumors. At later time point (day 56) there was no detectable virus in any normal organ (Fig. 4b). Collectively, these data suggest that CF33-GFP is safe in mice.

We also imaged the mice weekly to visualize virus-encoded GFP. By day 7, GFP was detectable in injected tumors of all mice but not in un-injected tumors (Fig. 4c). However, by day 14 all mice had detectable GFP signal in both of their tumors. Interestingly, by day 28, the mean fluorescence intensity in un-injected tumors exceeded that in virus-injected tumors (Fig. 4d).

### CF33-GFP shows anti-tumor effect against A549 xenografts at very low dose

**Fig. 5:**
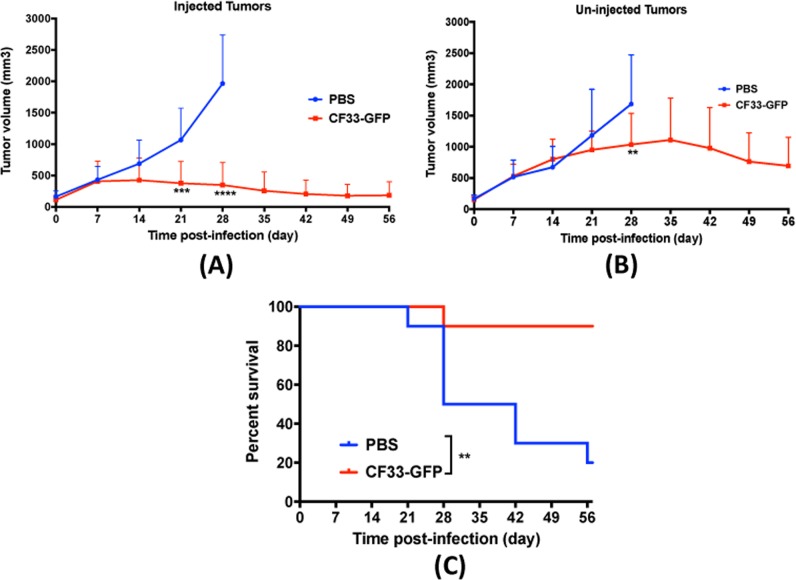
CF33-GFP shows anti-tumor efficacy in A549 tumor model. **a**, **b** Tumor volumes in mice treated in Fig. 4 were calculated using digital caliper. Each line represents volume for an individual tumor. Stats: two-way ANOVA; **P < 0.01, ***P < 0.005, ****P < 0.001. **c** Mice were euthanized when the volume of one of the two tumors exceeded 2500 mm^3^ and survival curve for the virus-treated group was compared with that of the PBS-treated group. Stats: Log-rank (Mantel Cox) test; **P < 0.01. Data pooled from two independent experiments (*n* = 5 + 5 per group)

In A549 xenograft model, a single intra-tumoral injection of very low dose of virus, i.e., 10^3^ PFUs, showed strong anti-tumor effect in injected tumors (Fig. 5a). Furthermore, CF33-GFP was able to disseminate to un-injected, distant tumors and showed anti-tumor effect in those tumors, especially during late time point (Fig. 5b). More importantly, virus treatment significantly increased the survival of mice compared to PBS-treated group (Fig. 5c). In the PBS treated group 8 out of 10 mice had to be euthanized due to tumor burden within 56 days after treatment whereas only one mouse in virus-treated group had to be euthanized during the study period. The mouse in virus-treated group was euthanized because the un-injected tumor reached maximum allowed tumor volume.

### CF33-GFP shows significant anti-tumor activity in a syngeneic mouse model of lung cancer

**Fig. 6:**
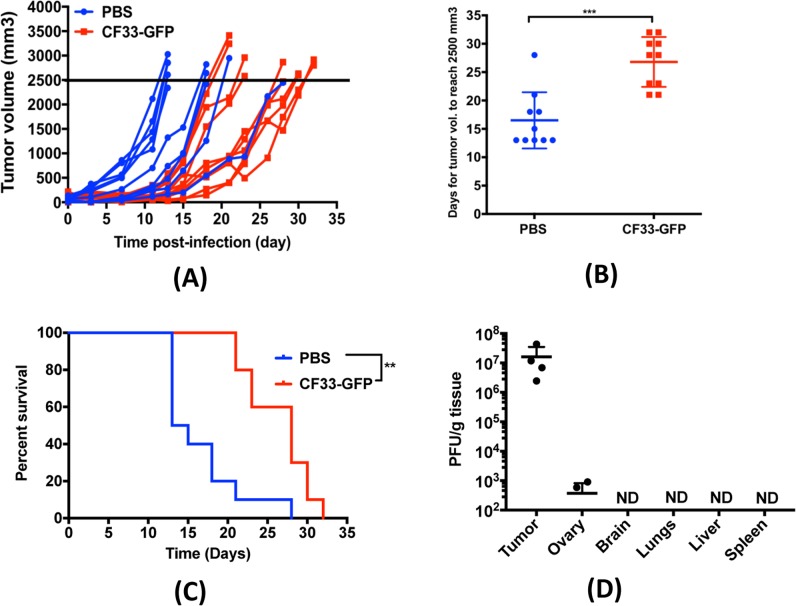
CF33-GFP shows anti-tumor efficacy in a syngeneic mouse model of lung cancer. **a** Immune-competent C57BL/6 mice bearing sub-cutaneous LLC1 tumor in the flank were given intra-tumoral injections on day 1, 3, and 5 (10^7^ PFU/injection) of CF33-GFP or PBS. **a** Tumor volumes were measured every other day; each line represents volume of a single tumor. Data pooled from two independent experiments (*n* = 5 + 5 per group). **b** Time (in days) for tumors to reach 2500 mm^3^ in volume, has been compared for the two treatment groups. Stats: student’s t-test; ***P < 0.005. **c** Mice were euthanized when their tumor volume exceeded 2500 mm^3^ and survival curve for the virus-treated group was compared with that of the PBS-treated group. **P < 0.01. **d** Mice were injected as in (**a**) and were euthanized (*n* = 4) 7 days after first injection. Virus titer in tumors and normal organs were determined by plaque assay. ND: not detected; Limit of detection: 100 pfu/g

C57BL/6 mice bearing sub-cutaneous LLC1 tumors (single tumor/mouse) were injected intra-tumorally with CF33-GFP or PBS. For this model, we used higher amount of virus and multiple injections (10^7^ PFUs/injection on day 1, 3, and 5) as our in vitro data showed that LLC1 cells are less sensitive to the virus. No sign of toxicity was observed in any mouse. While virus-treated tumors grew slower than PBS-treated tumors during early time point (up to 2 weeks), at later time points even the virus-treated tumors started growing rapidly (Fig. 6a). Nevertheless, overall growth of virus-treated tumors was slower compared to the PBS-treated tumors and time for the virus-treated tumors to reach 2500 mm3 in volume (maximum allowed tumor volume) was significantly higher than that for the PBS-treated tumors (Fig. 6b). Consequently, overall survival of mice in virus-treated group was significantly higher than the PBS-treated group (Fig. 6c).

We also performed bio-distribution studies at day 7 after first injection of virus. High titer of virus was detected in the tumors whereas no virus was detected in any normal organ except for ovary (Fig. 6d). Two out of four mice had detectable virus in their ovaries.

**Fig. 7:**
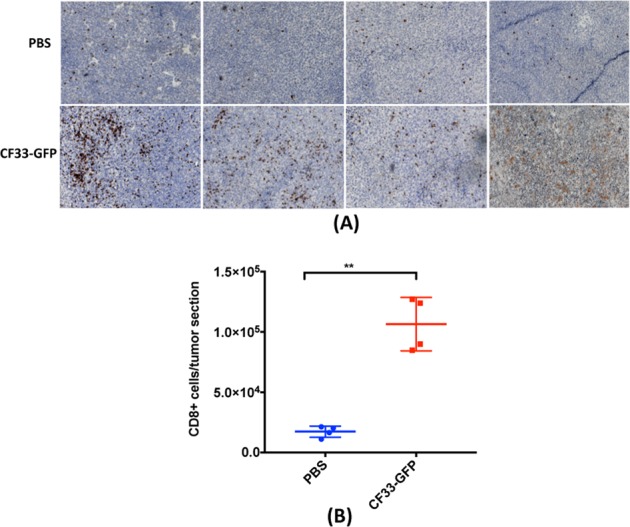
CF33-GFP increases CD8+ T cells infiltration in tumors. **a**, **b** Immune-competent mice bearing LLC1 tumors, treated as in Fig. 6, were euthanized (*n* = 4) on day 7 after first virus injection. **a** Tumor sections were stained for CD8+ T cells. Each section is from an individual mouse. Original magnification ×100. **b** CD8+ T cells were enumerated in all tumor sections using ImagePro software and were compared between virus-treated and PBS-treated groups. Stats: Student’s *t*-test; **P < 0.01

In a parallel study, we determined tumor infiltration by CD8+ T cells by immunohistochemical staining. Compared to the PBS-treated tumors, virus-treated tumors had significantly higher number of CD8+ T cells (Fig. 7a & b).

## Discussion

We have previously reported the construction of a chimeric poxvirus CF33 that is more efficient in killing cancer cells than the parental poxviruses [20, 21]. Sequence analysis of CF33 reveals that its genome is derived mainly from 3 VACV strains: IHD, Lister and WR. Together these three strains of VACV make about 77% of the 189,404 bp of CF33 genome, and most of the immune-modulating genes in CF33 are derived from them. The remaining 23% of CF33 genome is composed of the VACV strain AS, CL, LC as well as cowpox and rabbitpox. It is worth noting that the laboratory strain WR makes up the highest proportion of CF33 genome (~30.5%). A previous study by Thorne et al. [32] showed that among many different strains of VACVs, WR has the highest replication efficiency and cytotoxic potential in tumor cells. Interestingly, CF33 was found to have higher cytotoxic potential than all its parental viruses, including WR strain, in NCI-60 panel of cancer cell lines [20]. Very recently, Ricorde et al. [33] reported the generation of a chimeric poxvirus through recombination among 4 different VACV strains: WR, Wyeth, MVA and Copenhagen. The authors showed that the chimeric virus had higher cancer cell killing capacity and tumor selectivity compared to the parental VACV strains, in vitro.

In our previous studies we showed that CF33 exerts strong anti-tumor effect in pancreatic and colorectal cancer models at very low dose. Here, for the first time we report the genomic sequence analysis of CF33 and the oncolytic properties of *J2R*-deleted CF33 in lung cancer model using both xenograft and syngeneic tumor models. In accordance with other published studies on the cancer specificity of *J2R*-deleted poxviruses, CF33-GFP demonstrated higher replication and cell killing ability in cancer cells compared to normal cells in vitro [9, 34, 35]. Furthermore, like several other OVs, CF33-GFP was found to induce markers of ICD in lung cancer cells, in vitro [36–38]. This is consistent with our previous studies in pancreatic and colorectal cancer models [20, 21].

In vivo cancer specificity and safety of CF33-GFP is evident from the bio-distribution study in A549 xenograft model. Although high levels of virus were detected in tumors at early and late time points, in normal organs low levels of virus were detected only at early time point but not at late time point. Among the normal organs, ovaries had the highest titer of virus at day 7 in A549 xenograft model. Also, in the syngeneic LLC1 model two out of four mice had detectable virus at day 7 after virus injection. This, however, is not surprising as vaccinia virus has been known to preferentially replicate in ovaries [39, 40]. Interestingly, no virus was detected in the ovaries of nude mice 56 days after virus injection. This suggests that while the virus can disseminate to normal organs even after intra-tumoral injection, the virus is eventually cleared from those organs. Furthermore, a single injection of 10^3^ PFUs of the virus, a dose 100–1000 folds lower than that commonly reported for oncolytic poxviruses [41–44], showed significant anti-tumor effects in both injected and un-injected distant tumors leading to significantly improved survival of mice.

In LLC1 syngeneic tumor model, although the virus delayed tumor growth and significantly increased survival of the mice, anti-tumor efficacy of the virus was much less profound in this model than in the A549 xenograft model. There are several possible reasons for this; first, from our in vitro data it is clear that LLC1 supports very low levels of virus growth, and virus-mediated killing of LLC1 is much lower compared to human cancer cells. Hence, in vivo direct killing of LLC1 cells by the virus may have been minimal. Second, like most other mouse tumors, LLC1 tumors grow much more aggressively than tumors of human origin in mice [45, 46]. This is also evident from the fact that we used 5 × 10^6^ A549 cells but only 5 × 10^4^ LLC1 cells to generate each tumor. Despite using 100-fold less LLC1 cells, most of the PBS-treated LLC1 tumors reached the maximum tumor burden within 2 weeks whereas it took 3–5 weeks for the PBS-treated A549 tumors to reach similar volume. Third, in nude mice the virus can survive for much longer duration whereas in immune-competent mice immune cells can rapidly clear virus. Nevertheless, if the virus could appropriately activate anti-tumor immunity, there is a possibility that even if the virus is cleared earlier, the immune system can control tumor in syngeneic models. Our data show that CF33-GFP was able to increase tumor infiltration by CD8+ T cells, however this was not enough to control the aggressively growing LLC1 tumors. It is also likely that the infiltrated CD8+ T cells were blocked by check-point proteins such as PD-L1, in which case a combination of checkpoint inhibitor may enhance the anti-tumor efficacy of the virus. We plan to perform in depth studies on immune-modulation by the virus and combination of the virus with checkpoint inhibitors in future.

In summary, CF33-GFP shows cancer specificity in vitro and in mice. Furthermore, the virus is safe and exerts anti-tumor effects against both human and murine lung cancer, in mice. The overall anti-tumor efficacy of this virus may further be enhanced by combining with checkpoint inhibitors such as antibody against PD-L1 or other immune-modulatory therapeutics.
